# Supplementary material for: Prevalence, spatial distribution and determinants of infant mortality in Ethiopia: Findings from the 2019 Ethiopian Demographic and Health Survey
Source: PLoS One. 2023 Apr 25;18(4):e0284781. doi: 10.1371/journal.pone.0284781 (PMC10128957; doi:10.1371/journal.pone.0284781)
Supplement: Attachment — Submitted filename: Ethiopia paper review.pdf [file pone.0284781.s001.pdf]

I thank the editor for the chance to review this paper.

The authors present a novel study from Ethiopia where they have conducted secondary data analysis using the 2019 wave of the Ethiopian Demographic Health Survey to assess prevalent spatial determinants and distribution of infant mortality in the country. The context of the research is set in the high infant mortality burden region of sub-Saharan Africa, with a within known high mortality rate in Ethiopia. a need to produce updated findings and to share special determinants and distribution of infant mortality is provided as a rationale for the study. the authors use a variety of analyses to assess the determinants of infant mortality rate, which has been assessed mainly by logistic regression analysis, where the outcome of infant mortality as a binary variable is assessed across known social demographic factors. the spatial element is analysed using three different techniques ranging from spatial autocorrelation analysis hot spot analysis, and set scan statistical analysis. The key results observed for their regression analysis factors linked to maternal age, low antenatal care follows up, and residents in the Somali region while the spatial analysis results produced geographical details, their interpretation and results were limited.

The paper has issues in the presentation of the findings and linking the spatial analysis with the regression analysis to which I have identified comments, and I encourage the authors to address the substantive nature of these comments major however, considering the importance of the findings of the paper to understanding IMR in Ethiopia, I ask the authors to revisit the paper to address my comments:

Introduction section

Comment one: The opening paragraph of the introduction does not directly bring the audience to the research aim of this study. A definition to start off is not required it has value, but it needs to be provided later. while reference to the millennial development goal is made in the context of sub-Saharan Africa the authors should provide data of Ethiopia’s infant mortality rate before the MDG the progress in the MDG and why there is a gap

Overall comment for Introduction: The introduction section needs to be reworked to bring in more new relevant literature on the global IMR reduction, and Ethiopia's current infant mortality rate. Providing a summary of Ethiopia’s historical performance using the MDGs as a reference to explain why there is an IMR gap against the health system, policy and social factors is suggested. The authors need to contextualise Ethiopia in the context of Sub-Saharan Africa but additionally also to identify why the authors think that special distribution determinants are linked to infant mortality in the introduction the research gap for the study has not been adequately provided.

While some details of Ethiopia provided under study design and settings in the first paragraph those details need also to be linked to the contextual factors explaining high rates of IMR providing a running commentary of administrative details of Ethiopia is not informative to the reader to understand why what hard connects to IMR.

Comment 2;

Please revise the data source statement to use standard language in reference to accessing secondary data from the data owner.

Methods:

Comment3: In the methods, the authors do well to provide some details of the outcome variable; however, the authors may also like to provide a section on both outcome confounder variables and segregate the confounding variables across the various types, such as individual household community health system but As for any structure that the authors would like to use

Comment 4: Please provide a summary section that explains why the different types of spatial analysis were conducted and how the different types of spatial analysis provide better information to understand the infant mortality rate. Apart from spatial autocorrelation, the author's lead needs to provide rationale and evidence from the literature about the other spatial analysis methods used and why they have been additionally performed.

Comment 5 Interesting results are produced from the regression analysis and the spatial analysis the results and discussion do not seem to bring out the findings in a substantial manner or in a manner in which the results from each of the spatial methods bills onto each other.

Comment 6 ; Lastly, the paper's discussion study does not adequately provide insights to link back the methods, results and the research gap. I strongly advise the authors to consider working on the discussion section, which in its current form does not adequately draw out the results produced. The paper again is important in its contribution to the literature, but it needs more work in this section.

Comment 7 The limitation section needs to bring in more known limitations and types of bias with research that uses secondary data analysis and spatial analysis.

Comment 8. Please check the reference formatting (ref 13) for example has an incorrect author name.

Comment 9: All the spatial analysis figures are blurry and not of clear quality. Please provide clearer images.
