# Supplementary material for: Prevalence, spatial distribution and determinants of infant mortality in Ethiopia: Findings from the 2019 Ethiopian Demographic and Health Survey
Source: PLoS One. 2023 Apr 25;18(4):e0284781. doi: 10.1371/journal.pone.0284781 (PMC10128957; doi:10.1371/journal.pone.0284781)
Supplement: Attachment — Submitted filename: Rebuttal letter.docx [file pone.0284781.s002.docx]

**Response to comments**

PONE-D-22-21335

Prevalence, spatial distribution and determinants of Infant Mortality in Ethiopia: Findings from 2019 Ethiopian Demographic and Health Survey.

**Journal:** PLOS ONE

**Subject:** Submission of revised manuscript

We are very welcome for the reviews provided by the editor and reviewer of this manuscript. The comments are encouraging and the reviewer appears to share our judgment that this study and its results are scientifically important. Kindly see below, our detailed response to the comments. Also, kindly find the attached file of the revised manuscript that is uploaded separately.

Yours sincerely,

**Tadesse Tarik Tamir (on behalf of all authors)**

University of Gondar, Gondar, Ethiopia

**Response to editor comments**

1. Please ensure that your manuscript meets PLOS ONE's style requirements, including those for file naming. The PLOS ONE style templates can be found at 
<https://journals.plos.org/plosone/s/file?id=wjVg/PLOSOne_formatting_sample_main_body.pdf> and 
<https://journals.plos.org/plosone/s/file?id=ba62/PLOSOne_formatting_sample_title_authors_affiliations.pdf>

**Response:** Dear editor, thank you for the direction. The authors ensured for the manuscript meeting PLOS ONE's style requirements.

2. Thank you for submitting the above manuscript to PLOS ONE. During our internal evaluation of the manuscript, we found significant text overlap between your submission and previous work in the Methods section. We would like to make you aware that copying extracts from previous publications word-for-word is unacceptable. In addition, the reproduction of text from published reports has implications for the copyright that may apply to the publications. Please revise the manuscript to rephrase the duplicated text, cite your sources, and provide details as to how the current manuscript advances on previous work. Please note that further consideration is dependent on the submission of a manuscript that addresses these concerns about the overlap in text with published work. We will carefully review your manuscript upon resubmission and further consideration of the manuscript is dependent on the text overlap being addressed in full. Please ensure that your revision is thorough as failure to address the concerns to our satisfaction may result in your submission not being considered further.

**Response: D**ear editor, thank you for the careful evaluation of our manuscript. We have cross-checked the similarity of our manuscript with the previous publications. The same wording in our manuscript and other studies might be due to the similarity in methodology. We believe that the concern is addressed in our revised manuscript. The authors are keen to reconsider for any concerns that arise by the editor on the way of possible publication of the manuscript. Thank you!

3. PLOS requires an ORCID iD for the corresponding author in Editorial Manager on papers submitted after December 6th, 2016. Please ensure that you have an ORCID iD and that it is validated in Editorial Manager. To do this, go to ‘Update my Information’ (in the upper left-hand corner of the main menu), and click on the Fetch/Validate link next to the ORCID field. This will take you to the ORCID site and allow you to create a new iD or authenticate a pre-existing iD in Editorial Manager. Please see the following video for instructions on linking an ORCID iD to your Editorial Manager account: <https://www.youtube.com/watch?v=_xcclfuvtxQ>

Response: Dear editor, thank you for informing us of the ORCID iD requirement by PLOS ONE. The corresponding author authenticated a pre-existing iD as per requirement.

4. Your ethics statement should only appear in the Methods section of your manuscript. If your ethics statement is written in any section besides the Methods, please delete it from any other section.

**Response:** Dear editor, thank you for your guidance on the ethics statement of the manuscript. The authors have written the ethics statement only in the method section as per your direction. Kindly find the point on page number 7 of our updated manuscript.

5. We note that Figures 3 and 4 in your submission contain map images which may be copyrighted. All PLOS content is published under the Creative Commons Attribution License (CC BY 4.0), which means that the manuscript, images, and Supporting Information files will be freely available online, and any third party is permitted to access, download, copy, distribute, and use these materials in any way, even commercially, with proper attribution. For these reasons, we cannot publish previously copyrighted maps or satellite images created using proprietary data, such as Google software (Google Maps, Street View, and Earth). For more information, see our copyright guidelines: <http://journals.plos.org/plosone/s/licenses-and-copyright>.

**Response:** Dear editor, thank you for the concern. We believe that now we have addressed the issue. Kindly find the point in the caption of figures 3 and 4 in our updated manuscript.

With Kind Regards

**Response to reviewer comments**

**Reviewer #1**

Dear reviewer, First and foremost, we appreciate your enthusiasm for our manuscript's subject and hypotheses, as well as your detailed perspectives and insightful comments.

**Introduction section**

**Comment one:** The opening paragraph of the introduction does not directly bring the audience to the research aim of this study. A definition to start off is not required it has value, but it needs to be provided later. while reference to the millennial development goal is made in the context of sub-Saharan Africa the authors should provide data of Ethiopia’s infant mortality rate before the MDG the progress in the MDG and why there is a gap

**Overall comment for Introduction**: The introduction section needs to be reworked to bring in more new relevant literature on the global IMR reduction, and Ethiopia's current infant mortality rate. Providing a summary of Ethiopia’s historical performance using the MDGs as a reference to explain why there is an IMR gap against the health system, policy and social factors is suggested. The authors need to contextualise Ethiopia in the context of Sub-Saharan Africa but additionally also to identify why the authors think that special distribution determinants are linked to infant mortality in the introduction the research gap for the study has not been adequately provided.

While some details of Ethiopia provided under study design and settings in the first paragraph those details need also to be linked to the contextual factors explaining high rates of IMR providing a running commentary of administrative details of Ethiopia is not informative to the reader to understand why what hard connects to IMR.

**Response:** Dear reviewer, thank you for the guidance. Based on your feedback, we have considered a modification of the introduction to the current manuscript. Kindly find the section on page number 3, lines 2-30.

**Comment 2;**

Please revise the data source statement to use standard language in reference to accessing secondary data from the data owner.

**Response:** Dear reviewer, thank you for your guidance. We have added a statement that clarifies the section. Please find the point on page number 4-5.

**Methods:**

**Comment 3:** In the methods, the authors do well to provide some details of the outcome variable; however, the authors may also like to provide a section on both outcome confounder variables and segregate the confounding variables across the various types, such as individual household community health system but As for any structure that the authors would like to use.

**Response:** Dear reviewer, thank you for the concern. The authors checked for multicollinearity between explanatory variables using VIF with its value of less than ten (3.4) and used multiple variables (a better method for controlling for multiple confounding) in the final best-fitted model of regression. Kindly find the point on page 5, lines 9-11.

**Comment 4:** Please provide a summary section that explains why the different types of spatial analysis were conducted and how the different types of spatial analysis provide better information to understand the infant mortality rate. Apart from spatial autocorrelation, the author’s lead needs to provide rationale and evidence from the literature about the other spatial analysis methods used and why they have been additionally performed.

**Response:** Dear reviewer, thank you for the concern. We have addressed the comment accordingly. please find the point on page 5.

**Comment 5:** Interesting results are produced from the regression analysis and the spatial analysis the results and discussion do not seem to bring out the findings in a substantial manner or in a manner in which the results from each of the spatial methods bills onto each other.

**Response:** Dear reviewer, you are correct. We have seen the section carefully and done a kind of modification as per your concern.

**Comment 6**; Lastly, the paper’s discussion study does not adequately provide insights to link back the methods, results, and the research gap. I strongly advise the authors to consider working on the discussion section, which in its current form does not adequately draw out the results produced. The paper again is important in its contribution to the literature, but it needs more work in this section.

**Response:** Dear reviewer, thank you for the constructive feedback on the section which could help our manuscript scientifically sound. Taking your feedback into account, we have added additional statements to the discussion section in our updated manuscript based on your comment. Kindly find the points on page number 9-11.

**Comment 7** The limitation section needs to bring in more known limitations and types of bias with research that uses secondary data analysis and spatial analysis.

**Response:** Dear reviewer, thank you for the guidance. We added a statement in our current manuscript as per your direction. Please find the section on page number 11-12.

**Comment 8.** Please check the reference formatting (ref 13) for example has an incorrect author name.

**Response:** Dear reviewer, thank you for the feedback. We corrected the section accordingly.

**Comment 9:** All the spatial analysis figures are blurry and not of clear quality. Please provide clearer images.

**Response**: Comment accepted, and the figures have been reworked for clarity.

Once again, we thank you for your insightful comments, suggestions, and guidance, which have significantly improved the study.

**Reviewer #2**

Dear reviewer, first of all, we appreciate the enthusiasm for our manuscript's subject and hypotheses, as well as your insightful comments.

In ABSTRACT, in the conclusion subsection, the author stated that PNC coverage in clustered regions should be devised and strengthened. However, in their study, PNC coverage was not a significant predictor of infant mortality. The authors should emphasize what they found significant in their study. Moreover. The abbreviation PNC has not been defined elsewhere in this section; therefore, it is not clear what it represents. The statement was higher than the worldwide objective and is not clear. What is the objective worldwide? An appropriate term should be a goal or a target. The abbreviations should be expanded when used for the first time. (eg, PNC, EDHS, DHS).

**Response:** Dear reviewer, thank you for such a constructive comment. The authors have addressed the comments as per your direction. Kindly find the point on the section in our updated manuscript.

In result,

¨A little beat greater than half (51.38%) of the study participants were born from poor family (Table 1) ¨. Beat should be replaced with bit; however, the statement was not written in a formal way to write the article.

**Response:** Dear reviewer, appreciating your in-depth review of the manuscript we corrected the section accordingly.

The discussion section is inadequate.

**Response:** Dear reviewer, thank you for the feedback on the discussion. We added statements to the section in our updated manuscript based on your comment. Kindly find the points on page numbers 9-11.

In addition, there are several language and editorial issues.

**Response:** Dear reviewer, thank you for the concern, and as per your direction we thoroughly edited the language issues together with language experts who are working at the University of Gondar.

Thank you!
