# Supplementary material for: Prevalence, spatial distribution and determinants of infant mortality in Ethiopia: Findings from the 2019 Ethiopian Demographic and Health Survey
Source: PLoS One. 2023 Apr 25;18(4):e0284781. doi: 10.1371/journal.pone.0284781 (PMC10128957; doi:10.1371/journal.pone.0284781)
Supplement: Attachment — Submitted filename: Rebuttal letter.docx [file pone.0284781.s003.docx]

**Response to comments**

PONE-D-22-21335

Prevalence, spatial distribution and determinants of Infant Mortality in Ethiopia: Findings from 2019 Ethiopian Demographic and Health Survey.

**Journal:** PLOS ONE

**Subject:** Submission of revised manuscript

We sincerely appreciate the editor's and reviewer's comments on this work. Kindly find our response to the comments below. Please refer to the attached file, which was uploaded independently and contains the revised manuscript.

Yours sincerely,

**Tadesse Tarik Tamir (on behalf of all authors)**

University of Gondar, Gondar, Ethiopia

Response to Editor comments

Please review your reference list to ensure that it is complete and correct. If you have cited papers that have been retracted, please include the rationale for doing so in the manuscript text, or remove these references and replace them with relevant current references. Any changes to the reference list should be mentioned in the rebuttal letter that accompanies your revised manuscript. If you need to cite a retracted article, indicate the article’s retracted status in the References list and also include a citation and full reference for the retraction notice.

**Response:** Dear editor, thank you for letting us know the PLOS ONE requirement regarding the reference list. We have reviewed our references and we have not used retracted references.

PLOS ONE does not copyedit accepted manuscripts, so the language in submitted articles must be clear, correct, and unambiguous. Any typographical or grammatical errors should be corrected at revision, so please note any specific errors here.

**Response:** Dear editor, thank you for your suggestions. We thoroughly made corrections to typographical or grammatical errors in the manuscript.

**Response to reviewer comments**

**Reviewer #4**

Dear reviewer, First and foremost, we appreciate your enthusiasm for our manuscript's subject and hypotheses, as well as your detailed perspectives and insightful comments.

1. As you know the abbreviations that come for the first time in the text should be stated completely. (ANC and PNC in abstract).

**Response:** Dear reviewer, you are correct abbreviations that come for the first time in the text should be stated completely. The concern is addressed accordingly.
2. Are there any exclusion criteria for this survey?

**Response:** Dear reviewer, thank you for the concern. For the survey on which our analysis was done, there are no declared exclusion criteria that could have an effect on our results.
3.I Recommend to use the recent literature in disscution.

**Response:** Dear reviewer, thank you for such a recommendation that makes our manuscript scientifically sound. We replaced some references in the discussion with more recent ones as per your recommendation. We used more recent references in our manuscript if they were otherwise not available with the best of our search.
4. According to the findings, it would be better that you make some recommendations at the end of discussion.

**Response:** Dear reviewer, thank you for such an insightful comment. We made a kind of recommendation at the end of the discussion as per your direction. Please find the point at the end of the discussion in our revised manuscript.
5. I recommend to clarify the findings in figure 2 in the result part, such as critical values.

**Response:** Dear reviewer, we made modifications to the section as per your recommendation. Kindly find the point in the result part of the revised manuscript.
6. The map and graphs are well designed.

**Response**: Dear reviewer, we are overjoyed to have such positive feedback on our work from such esteemed reviewers as you.
